# The Relationship of Eating Rate and Degree of Chewing to Body Weight Status among Preschool Children in Japan: A Nationwide Cross-Sectional Study

**DOI:** 10.3390/nu11010064

**Published:** 2018-12-29

**Authors:** Hitomi Okubo, Kentaro Murakami, Shizuko Masayasu, Satoshi Sasaki

**Affiliations:** 1 Department of Health Promotion, National Institute of Public Health, Saitama 351-0197, Japan; 2 Department of Social and Preventive Epidemiology, School of Public Health, University of Tokyo, Tokyo 113-0033, Japan; kenmrkm@m.u-tokyo.ac.jp (K.M.); stssasak@m.u-tokyo.ac.jp (S.S.); 3 Ikurien-Naka, Ibaraki 311-0105, Japan; masayasu@ikurien.com

**Keywords:** rate of eating, degree of chewing, weight status, cross-sectional study, preschool children

## Abstract

There is growing recognition that eating slowly is associated with a lower risk of obesity, and chewing well might be an effective way to reduce the eating rate. However, little is known about these relationships among children. We therefore investigated the associations of eating rate and chewing degree with weight status among 4451 Japanese children aged 5–6 years. Information on eating rate (slow, medium, or fast), degree of chewing (not well, medium, or well), and nutrient intake of children were collected from guardians using a diet history questionnaire. Weight status was defined using the International Obesity Task Force cut-offs based on BMI calculated from guardian-reported height and weight. The prevalence of overweight and thinness was 10.4% and 14.3%, respectively. A higher eating rate and a lower degree of chewing were associated with being overweight (both *p* < 0.001). Eating slowly was associated with being thin (*p* < 0.001), but no association was observed between chewing degree and thinness. These associations were still evident after controlling for potential confounders including parental educational attainment, weight status, and the child’s nutrient intake. In conclusion, this cross-sectional study suggested that chewing well, rather than eating slowly, might be a more effective way for healthy weight management among Japanese preschool children.

## 1. Introduction

The increasing prevalence of overweight and obesity in children is widely recognized as a global public health challenge [1]. Great attention has therefore been focused on the critical period in the life course when the risk of obesity is increased and when nutritional and other lifestyle interventions may be more effective in preventing excess weight gain [2,3]. There is considerable evidence that early childhood is an important period for determining the future risk of obesity and its related adverse consequences [4,5]. Since eating behaviors emerge and are established in early life, and may persist into adulthood [6,7], understanding potential modifiable eating behaviors that influence excess weight gain may help in the development of intervention strategies to tackle childhood obesity [7,8].

Among eating behaviors, the rate of eating is now beginning to receive considerable scientific interest. A recent meta-analysis of observational studies among adults clearly showed that eating quickly was associated with increased body mass index (BMI) and a higher prevalence of obesity [9]. Similar positive associations have also been observed in children [10,11,12,13,14,15]. These findings raise the possibility that reducing the eating rate may be beneficial in terms of weight control [16]. Various methods may be available to reduce eating rate such as introducing within-meal pauses, decreasing the amount of food in each mouthful, using computerized devices, or a combination of these methods [17]. Chewing one’s food well can be an effective and easy way to reduce eating rate, and might contribute to a lower risk of obesity [17,18]. Several studies have actually suggested that chewing slowly or increasing the number of chewing cycles during meals is associated with a lower BMI [19,20,21]. However, the findings remain inconclusive, and no studies among young children have been conducted. Additionally, it should be noted that while almost all studies have exclusively examined the relationship between eating behaviors and the risk of obesity [9], little is known about the relationship to the risk of thinness [22]. Given that the prevalence of thinness has recently increased among Japanese adolescents [23,24], it is necessary to address the potential contributions of eating rate and chewing degree to not only the risk of being overweight, but also to that of thinness. 

Here, using data from the Japan Nursery School SHOKUIKU Study [25,26], we examined the hypothesis that eating rate and chewing degree are associated with weight status in a group of Japanese preschool children, taking into account a range of potential confounding factors including parental educational attainment, weight status, and the child’s nutrient intake.

## 2. Materials and Methods

### 2.1. Study Population and Procedure

The Japan Nursery School SHOKUIKU Study was a nationwide, cross-sectional, self-administered questionnaire survey conducted in 44 of 47 prefectures in Japan. The main objective of the SHOKUIKU Study was to investigate the nutritional and health status and dietary habits of preschool children, and to collect basic information on attitude and demands related to nutrition education (shokuiku) from the children’s guardians, dietitians, and nursery school teachers. The study design, subject recruitment, and study procedure have been previously described in detail [25,26]. In brief, a set of two self-administered questionnaires on dietary habits and lifestyle behaviors of children were distributed to 9762 guardians of children aged 5 or 6 years in 416 nursery schools via dietitians or school governesses, and collected between February and March 2011. The number of respondents was 5637 (response rate = 57.7%) for the lifestyle questionnaire and 6256 (64.1%) for the diet history questionnaire. A total of 5514 guardians of children completed both questionnaires (response rate = 56.5%). For analysis, we excluded children whose guardians answered “not sure” or who had missing data for eating rate (*n* = 89) or chewing degree (*n* = 35), those who had missing information on the other variables used (*n* = 895), those who were currently receiving dietary counselling from a doctor or dietician (*n* = 64), or those whose energy intake was extremely low (<500 kcal/d) or high (>3000 kcal/d) (*n* = 28). As some children were in more than one exclusion category, the final analysis comprised 4451 children from 374 nursery schools.

The study protocol was approved by the ethics committee of the Japanese Society of Nutrition and Dietetics (approval number 2010_09_01). Responding to the questionnaire was regarded as agreement to participate in this study, in accordance with the Ethical Guidelines for Epidemiological Research established by the Ministry of Health, Labor, and Welfare.

### 2.2. Eating Behaviors

Rate of eating was assessed by the response to the following question: “How fast is your child’s rate of eating compared with that of other children?”, chosen from six qualitative categories, namely “very slow”, “relatively slow”, “medium, “relatively fast”, “very fast”, and “not sure”. The question for degree of chewing was “How well does your child chew foods while eating?”. The response was chosen from six qualitative categories, namely “very well”, “relatively well”, “medium”, “not relatively well’, “not well”, and “not sure”. In the present study, we excluded subjects who responded with “not sure” as above-mentioned.

### 2.3. Anthropometric Measurements

Information on the children’s body weight and height was reported by their guardians. BMI (kg/m^2^) was calculated as weight (kg) divided by height (m) squared. The BMI z-score was computed using sex- and age-specific LMS values from the International Obesity Task Force (IOTF) [27]. Weight status was defined using the IOTF cut-offs for children based on sex- and age-specific BMI [27]. Children with BMI values that corresponded to an adult BMI <18.5 kg/m^2^ were classified as thin, ≥18.5 to <25 kg/m^2^ as normal weight, and ≥25 kg/m^2^ as overweight.

### 2.4. Covariates: Child Lifestyle Variables

We collected information on the following variables through a four-page questionnaire that was designed for this study: residential area, paternal and maternal educational attainment, maternal and paternal body weight and height, child’s birthweight, and number of siblings. Parental weight status was defined based on the BMI calculated from self-reported height and weight as follows: underweight (<18.5 kg/m^2^), normal (≥18.5 to <25.0 kg/m^2^), and overweight (≥25.0 kg/m^2^) [28]. The dietary habits of children over the preceding month were assessed using a brief-type diet history questionnaire for preschool children (BDHQ3y) [29]. The BDHQ3y is a four-page, structured questionnaire that inquiries about the consumption frequency of selected foods commonly consumed in Japan, general dietary behaviors, and usual cooking methods. It was developed based on comprehensive [30,31] and brief versions [32,33] of a validated self-administered diet history questionnaire for Japanese adults. Estimates of daily intake of foods (69 food items in total), energy, and selected nutrients were calculated using an ad hoc computer algorithm for the BDHQ3y, which was based on the Standard Tables of Food Composition in Japan [34]. In BDHQ3y, information on portion sizes was not collected; an age-specific fixed portion size was used in the calculations for all subjects. In the present study, values of nutrient intake were energy-adjusted using the density method (i.e., percentage of energy for macronutrients and amount per 1000 kcal of energy for dietary fiber). The relative validity of the BDHQ3y was confirmed through a comparison with 3-nonconsecutive-day diet records (DRs) among the guardians of 61 children aged 3–4 years [29]. Briefly, the median Pearson correlation coefficients between BDHQ3y and DR for energy-adjusted nutrient intake by density method were 0.32 for protein, 0.27 for total fat, 0.27 for carbohydrate, and 0.44 for dietary fiber [29]. Information on a child’s physical activity was also obtained from the BDHQ3y.

### 2.5. Statistical Analyses

All statistical analyses were performed using SAS statistical software version 9.4 (SAS Institute, Inc. Cary, NC, USA). Descriptive data are presented as means (95% confidence intervals) for continuous variables and percentages of subjects for categorical variables. Children were regrouped into three categories according to rate of eating (slow, medium, or fast) and degree of chewing (not well, medium, or well) because of the small number of cases in each of the extreme categories (“very slow” [*n* = 516] and “very fast” [*n* = 95] for the rate of eating and “not well” [*n* = 145] and “very well” [*n* = 160] for degree of chewing). Differences in group characteristics according to categories of eating rate and degree of chewing were examined using the Mantel–Haenszel χ^2^ test for categorical data and a linear trend test for continuous data.

Crude and multivariate-adjusted mean values of BMI z-score across categories of eating rate and degree of chewing were calculated using a general linear model. Logistic regression analysis was used to calculate the crude and multivariate-adjusted odds ratio (OR) and 95% CI for being overweight and thin against being normal weight for each category of eating rate and degree of chewing. The medium category of each eating behavior was used as a reference group. Multivariate models were adjusted for the following potential confounders: child factors were sex (boy or girl), age (months), number of siblings (0, 1, or ≥2), physical activity (low, middle, or high) and birthweight (<2500, 2500–3999, or ≥4000 g); family-related factors were residential block (Hokkaido and Tohoku; Kanto; Hokuriku and Tokai; Kinki; Chugoku and Shikoku; or Kyushu and Okinawa), paternal and maternal educational attainment (junior high school/high school, junior college/vocational technical school, or university), and paternal and maternal weight status (underweight, normal, or overweight) (multivariate model 1). We also conducted analyses with further adjustment for nutrient intakes including protein (% of energy), fat (% of energy), and dietary fiber (g/1000 kcal) (multivariate model 2). Tests for trend associations were assessed by a logistic regression model assigning consecutive integers (1–3) to the levels of the independent variables. In the present study, rate of eating was weakly correlated with degree of chewing (Spearman correlation coefficients r = −0.20, *p* < 0.001). We therefore investigated the independent associations between weight status and rate of eating and degree of chewing by mutual adjustment in multivariate models.

All reported *p* values are two-tailed, and *p* < 0.05 was considered to be statistically significant.

## 3. Results

### 3.1. Characteristics of Study Population

Characteristics of the 4451 analytical subjects are shown in Table 1. Compared with other participants in the SHOKUIKU Study (*n* = 1932), those included in these analyses were more likely to live in the Kanto, Hokuriku and Tokai or Kinki areas, to have parents with a higher educational attainment and normal body weight status, to have many siblings, to be born at a normal birthweight (2500–3999 g), to have a higher protein intake, and to have a lower carbohydrate intake (all *p* < 0.05). There were no differences in sex, age, physical activity, body height and weight, or in intake of energy, fat, and dietary fiber between the children studied and the remaining children.

When the children were classified into three groups according to rate of eating, the children who ate faster were more likely to be boys, to be older, to be physically active, to have higher body height and weight, to have mothers with a higher educational attainment, to have many siblings, to be born at a normal birthweight (2500–3999 g), to have higher intakes of energy and carbohydrate, and to have a lower intake of total fat (all *p* < 0.001). The parents whose children ate faster were more likely to be overweight (both *p* < 0.05). In contrast, the children who chewed their foods well were more likely to be girls, to be physically active, to have lower body height and weight, and to have higher intakes of protein and dietary fiber (all *p* < 0.001). Additionally, the mothers whose children chewed well were less likely to be overweight (*p* < 0.001).

### 3.2. Associations of Eating Rate and Degree of Chewing with BMI z-Score

Table 2 shows the associations of eating rate and chewing degree with BMI z-score. Rate of eating was positively associated with BMI z-score (*p* < 0.001). The positive associations remained after adjustment for potential confounding factors including sex, age, residential block, parental educational attainment, parental body weight status, number of siblings, physical activity, birthweight status, and dietary intake (both *p* < 0.001 in multivariate models 1 and 2). In contrast, degree of chewing was negatively associated with the BMI z-score irrespective of adjustment for confounding factors (all *p* < 0.001).

### 3.3. Associations of Eating Rate and Degree of Chewing with Weight Status

According to the IOTF cut-offs, the prevalence of overweight and thinness in this population were 10.4% and 14.3%, respectively. A clear positive association was observed between the rate of eating and the prevalence of overweight irrespective of adjustment for potential confounders (OR per one-category increase in rate of eating = 2.23 [95% CI: 1.93, 2.58], *p* for trend < 0.001) (Table 3). Compared to the children in the medium eating rate group, those in the slow group had a significantly lower prevalence of overweight, while those in the fast group had a significantly higher prevalence of overweight. The degree of chewing was negatively associated with being overweight (OR per one-category increase in degree of chewing = 0.48 [95% CI: 0.40, 0.57], *p* for trend < 0.001). The children who did not chew well had a significantly higher prevalence of overweight, whereas those who chewed well had a significantly lower prevalence of overweight when compared with the reference group.

When we examined the relationship between rate of eating and the prevalence of thinness (Table 4), rate of eating was negatively associated with being thin in all models (all *p* for trend <0.001). Compared to the children in the medium eating rate group, those in the fast group had a significantly lower prevalence of thinness, but those in the slow group had a significantly higher prevalence of thinness. In contrast, no association was observed between degree of chewing and prevalence of thinness.

We investigated the independent associations of eating rate and degree of chewing with weight status by mutual adjustment in multivariate models. The positive association of eating rate with the prevalence of overweight (OR = 2.03 [95% CI: 1.75, 2.35], *p* < 0.01) and the negative association of eating rate with the prevalence of thinness (OR = 0.60 [95% CI: 0.52, 0.69], *p* < 0.01) were independent of the chewing degree. Similarly, associations of chewing degree with the prevalence of overweight (OR = 0.56 [95% CI: 0.46, 0.68], *p* < 0.01) and that of thinness (OR = 0.99 [95% CI: 0.86, 1.16], *p* = 0.94) were not confounded by the eating rate.

In additional analyses, we evaluated the boys and girls separately and observed similar results (data not shown). We also checked the robustness of our findings by using the original five categories of eating rate and degree of chewing. Similar patterns of associations of eating rate and degree of chewing with weight status were observed (OR for being overweight per one-category increase in rate of eating = 2.04 [95% CI: 1.82, 2.30], *p* for trend < 0.001; OR for being thin per one-category increase in rate of eating = 0.66 [95% CI: 0.60, 0.74], *p* for trend < 0.001; OR for being overweight per one-category increase in degree of chewing = 0.56 [95% CI: 0.49, 0.65], *p* for trend < 0.001; and OR for being thin per one-category increase in degree of chewing = 1.10 [95% CI: 0.97, 1.23], *p* for trend = 0.13).

## 4. Discussion

In the present cross-sectional study, we focused on the rate of eating and degree of chewing since they are believed to influence body weight status. A higher rate of eating and a lower degree of chewing were significantly associated with a higher prevalence of overweight among Japanese preschool children. We also examined the relationship between these eating behaviors and the prevalence of thinness. Eating slowly was associated with a significantly higher prevalence of thinness, whereas no association was observed between the degree of chewing and that of thinness. Thus, our hypothesis was partly supported by the present study. The differences in body weight status were associated with the variations in rate of eating and degree of chewing, suggesting that the acquisition of healthy eating behaviors has important implications in the prevention of childhood thinness and obesity.

Our findings that the rate of eating was positively associated with the BMI z-score and the prevalence of overweight among preschool children were highly consistent with a growing body of evidence from adults [9], adolescents [10,11,12,13], and young children [14,15,16]. The increasing recognition that eating fast is associated with excess body weight has highlighted the role of mastication as one of the potential determinants in reducing the eating rate [17,18], but to date, few studies have examined the relationship between chewing behaviors and body weight status [17,19,20,21]. One observational study of 93 Japanese residents aged 35–61 years showed a negative association between the number of chewing cycles before swallowing and a risk of body weight increments of more than 10 kg [19]. Another US study of fully dentate healthy adults showed that overweight/obese participants used fewer chewing cycles and exhibited a shorter chewing duration than normal-weight participants did, and also found negative correlations between these chewing behaviors and BMI [20]. Similar negative correlations were also reported from an experimental study of young Japanese university students [21]. Our results, together with those of previous studies, showed a significant negative association between degree of chewing and prevalence of overweight, notwithstanding the differences in study design, study population, generation, and more importantly, the definition of chewing behaviors. Given that research examining the relationship between chewing behavior and weight status is very limited and still inconclusive, further prospective and intervention studies are needed to confirm these findings, especially among children, and to determine the importance of chewing for the prevention of obesity by reducing the eating rate.

A number of potential mechanisms have been suggested to link eating behaviors and weight status. One plausible mechanism for explaining the association between the rate of eating and excess body weight might be due to energy intake, as we observed that the children who ate fast had a higher energy intake than did those who ate slowly (Table 1). This could be supported by a systematic review of controlled experimental studies showing that a slower eating rate is associated with a lower energy intake in comparison to a faster eating rate [35], and this may be because fast eaters consume more foods before the brain recognizes the satiety signal [16]. On the other hand, a recent meta-analysis of observational studies implied the presence of a mechanism other than energy intake [9], and suggested the presence of a decrease in mastication in the fast eaters. The relation of mastication with the rate of eating and excess weight might be plausible, as we observed that the children who chewed well were more likely to eat slowly, and had a significantly lower BMI z-score and prevalence of overweight regardless of energy intake. The potential mechanism linking degree of chewing and a lower prevalence of overweight might be explained by dietary fiber [36]. Intake of dietary fiber was negatively associated with not only the degree of chewing (Table 1), but also the BMI z-score, irrespective of adjustment for the degree of chewing (BMI z-score per 1 g/1000 kcal increase in dietary fiber: β = −0.03 [95% CI: −0.06, −0.01], *p* = 0.01 in multivariate model 2 in Table 2). This suggests that a higher intake of dietary fiber might be both directly and indirectly associated with a lower BMI z-score through an increase in the amount of chewing. Other potential mechanisms might also be involved [18,37] and thus further studies are needed to clarify the biological processes that underlie these associations.

Although numerous epidemiological studies have examined the relationship between eating rate and obesity, to our knowledge, only one previous study has investigated the relationship between eating slowly and the prevalence of being underweight [22]. In a cross-sectional study of 2641 Japanese adolescents aged 12–13 years, adolescents who ate slowly were more likely to be underweight than were those who did not eat slowly. In keeping with the findings from the previous study [22], we also observed a negative association between the rate of eating and the prevalence of thinness. The children who ate slowly had a significantly higher prevalence of thinness in comparison with those in the reference group, while the children who ate fast had a significantly lower prevalence of thinness. In contrast, we found no association between the degree of chewing and the prevalence of thinness. It is not clear why the relationship of the prevalence of thinness differed between the rate of eating and degree of chewing, but it might be due, at least partly, to energy intake as we observed (Table 1) and discussed above [16,35]. Additionally, other intra-individual factors (e.g., satiety responsiveness and food cue responsiveness) [16,37] and/or environmental factors (e.g., limited meal time and family-related factors) [38], which relate to reduced eating rate, might also be involved. Further investigations are needed to identify factors that are related to the rate of eating and to clarify the mechanisms leading to the differences in the relationship to the prevalence of thinness among eating behaviors.

The strengths of the current study include its large number of subjects from a nationwide survey of Japan and our consideration of a range of confounding influences on the relationships of eating rate and degree of chewing with body weight status. However, the study did have some limitations. First, the study participants were not randomly selected from the general population of preschool children, and were volunteers, and thus relatively more health conscious. Thus, the present findings should not be fully generalized. Second, due to the cross-sectional design of the present study, we cannot exclude the possibility of reverse causation. However, the significant trends in the associations, suggestive of a “dose-response effect”, and the consistency of our findings for being overweight with those of recent prospective [39,40] and intervention studies [41,42] support a causal interpretation. Third, the eating rate and degree of chewing of children were guardian reported, and the validity has not been examined. Thus, more valid measures of eating rate and degree of chewing are needed. Fourth, we applied BMI as a surrogate marker of adiposity, but a high BMI can reflect an increase in not only fat mass, but also fat-free mass [43]. A direct assessment of fat mass such as dual energy x-ray absorptiometry may be required. More importantly, BMI was calculated from guardian-reported body height and weight. Even though more than 95% of guardians in the present study checked the anthropometric data measured at nursery schools every month and confirmed the weight status of their children, this information might still be biased [44]. Finally, a higher rate of eating and a lower degree of chewing may act as a marker for other less healthy behaviors and obesogenic family influences, which could potentially confound associations with BMI in children. While we controlled for a range of potential confounding factors of BMI in children such as parental educational attainment, parental body weight status, and number of siblings, we cannot rule out unmeasured or residual confounding factors in this observational study. Additionally, we cannot account for measurement errors in the covariates considered.

## 5. Conclusions

This nationwide cross-sectional study in a group of Japanese preschool children showed that eating slowly was independently associated with not only a lower prevalence of overweight, but also a higher prevalence of thinness. In contrast, chewing well was associated with a lower prevalence of overweight, but not of thinness. Chewing well, rather than eating slowly, might be a more effective and practical way to control healthy weight status among Japanese preschool children. Further prospective and intervention studies are needed to confirm these findings and to determine the importance of eating behaviors established in early life for the prevention of childhood thinness and obesity.

## Figures and Tables

**Table 1:** Characteristics of subjects according to rate of eating and degree of chewing among Japanese preschool children ^a^.

	Total		Rate of Eating						*p* Trend ^b^	Degree of Chewing						*p* Trend ^b^
			Slow		Medium		Fast			Not Well		Medium		Well		
	(*n* = 4451)		(*n* = 919)		(*n* = 1908)		(*n* = 624)			(*n* = 954)		(*n* = 2874)		(*n* = 623)		
	Mean	SD	Mean	SD	Mean	SD	Mean	SD		Mean	SD	Mean	SD	Mean	SD	
Sex (%)																
Boy	52.4		46.1		54.8		65.1		<0.001	64.1		51.0		41.7		<0.001
Girl	47.5		53.9		45.2		34.9			36.0		49.0		58.3		
Age (months)	76.7	3.5	76.2	3.5	77.0	3.5	77.4	3.5	<0.001	76.9	3.5	76.7	3.5	76.6	3.5	0.07
Body height (cm)	115.2	5.5	113.8	5.3	115.7	5.4	117.7	5.2	<0.001	116.1	5.8	115	5.5	114.4	5.0	<0.001
Body weight (kg)	20.7	3.2	19.6	2.5	20.9	3.0	22.9	4.0	<0.001	21.5	3.9	20.5	3.0	19.9	2.6	<0.001
Residential block (%)																
Hokkaido and Tohoku	13.1		14.4		12.2		11.9		0.65	13.7		12.7		14.1		0.69
Kanto	21.2		21.6		21.1		20.7			18.7		22.1		21.2		
Hokuriku and Tokai	24.8		23.7		25.2		26.8			26.8		24.7		21.8		
Kinki	12.1		11.3		12.2		14.3			13.4		11.4		13.2		
Chugoku and Shikoku	14.4		13.6		15.2		14.6			14.3		14.4		14.5		
Kyushu and Okinawa	14.4		15.4		14.3		11.9			13.1		14.7		15.3		
Paternal educational attainment (%)																
High school	50.7		50.7		51.5		48.4		0.31	49.3		51.7		48.5		0.61
Junior college or vocational technical school	19.4		20.0		18.8		19.1			19.0		19.0		21.7		
University	29.9		29.3		29.7		32.5			31.8		29.3		29.9		
Maternal educational attainment (%)																
High school	40.2		42.1		39.8		35.6		0.008	41.2		40.0		39.7		0.71
Junior college or vocational technical school	44.0		42.5		44.7		46.3			42.0		44.7		43.5		
University	15.8		15.4		15.5		18.1			16.8		15.3		16.9		
Paternal weight status (%)																
Underweight (BMI <18.5 kg/m^2^)	2.9		3.2		2.8		1.9		0.005	3.4		2.6		3.4		0.28
Normal (BMI 18.5–24.9 kg/m^2^)	70.3		71.6		69.7		68.1			68.1		70.9		70.8		
Overweight/obese (BMI ≥25.0 kg/m^2^)	26.9		25.2		27.5		30.0			28.5		26.5		25.8		
Maternal weight status (%)																
Underweight (BMI <18.5 kg/m^2^)	15.8		17.2		14.7		14.7		0.03	14.9		15.5		18.3		0.02
Normal (BMI 18.5–24.9 kg/m^2^)	75.2		73.7		77.2		73.6			74.7		75.6		73.8		
Overweight (BMI ≥25.0 kg/m^2^)	9.0		9.1		8.1		11.7			10.4		8.8		7.9		
Number of siblings (%)																
0	14.5		17.5		11.7		13.8		<0.001	16.6		13.5		16.1		0.11
1	53.6		53.6		53.8		53.0			56.0		52.8		53.8		
≥2	31.9		28.9		34.4		33.2			27.5		33.7		30.2		
Physical activity (%)																
Low	4.4		6.2		2.7		4.3		<0.001	7		3.8		3.4		<0.001
Middle	20.7		24.9		17.8		16.5			22.5		20.9		16.7		
High	74.9		68.9		79.6		79.2			70.4		75.3		70.9		
Birthweight (%)																
<2500 g	10.7		12.6		9.4		8.8		<0.001	9.8		10.7		12		0.16
2500–3999 g	88.3		86.8		89.5		89.3			89.3		88.2		87.2		
≥4000 g	1.0		0.6		1.1		1.9			0.9		1.1		0.8		
Energy intake (kcal/d)	1134	305	1120	304	1137	302	1171	313	<0.001	1132	292	1132	304	1148	329	0.38
Nutrient intake																
Protein (% of energy)	13.1	1.8	13.2	1.8	13.1	1.8	13.0	1.7	0.12	13	1.8	13.1	1.7	13.4	1.9	<0.001
Fat (% of energy)	28.1	4.7	28.5	4.8	27.9	4.7	27.6	4.7	<0.001	28.2	5.0	28.1	4.6	27.8	4.9	0.15
Carbohydrate (% of energy)	57.4	5.6	57.0	5.7	57.6	5.6	57.9	5.4	<0.001	57.4	5.9	57.4	5.4	57.4	5.9	0.89
Dietary fiber (g/1000 kcal)	5.3	1.2	5.3	1.1	5.3	1.2	5.2	1.1	0.16	5.2	1.2	5.3	1.1	5.5	1.2	<0.001

^a^ Values are means and SD for continuous variables and the percentage of participants for categorical variables. ^b^ A linear trend test was used for continuous variables; a Mantel–Haenszel chi-square test was used for categorical variables.

**Table 2:** Relationship of eating rate and degree of chewing with BMI z-score among Japanese preschool children.

	*n*	Crude ^a^		Multivariate Model 1 ^b^		Multivariate Model 2 ^c^	
		Mean	95% CI	Mean	95% CI	Mean	95% CI
Rate of eating							
Slow	1919	−0.21	(−0.25, −0.16)	−0.22	(−0.26, −0.17)	−0.21	(−0.26, −0.17)
Medium	1908	0.08	(0.03, 0.12)	0.08	(0.04, 0.13)	0.08	(0.04, 0.13)
Fast	624	0.54	(0.44, 0.63)	0.54	(0.45, 0.62)	0.53	(0.45, 0.61)
Effect per change in category		0.34	(0.30, 0.39)	0.36	(0.31, 0.40)	0.35	(0.31, 0.40)
*p* for trend		<0.001		<0.001		<0.001	
Degree of chewing							
Not well	954	0.19	(0.11, 0.27)	0.19	(0.12, 0.25)	0.18	(0.12, 0.25)
Medium	2874	0.00	(−0.04, 0.04)	0.00	(−0.04, 0.04)	0.00	(−0.04, 0.04)
Well	623	−0.17	(−0.25, −0.09)	−0.16	(−0.24, −0.08)	−0.16	(−0.24, −0.07)
Effect per change in category		−0.18	(−0.24, −0.13)	−0.17	(−0.23, −0.12)	−0.17	(−0.23, −0.12)
*p* for trend		<0.001		<0.001		<0.001	

^a^ Values for the BMI z-score are means and 95% CIs in parentheses. ^b^ Multivariate model 1 was adjusted for sex (boy or girl), age (months, continuous), residential block (Hokkaido and Tohoku; Kanto; Hokuriku and Tokai; Kinki; Chugoku and Shikoku; or Kyushu and Okinawa), paternal educational attainment (high school, junior college/vocational technical school, or university), maternal educational attainment (high school, junior college/vocational technical school, or university), paternal weight status (underweight, normal, or overweight), maternal weight status (underweight, normal, or overweight), number of siblings (0, 1, or ≥2), physical activity (low, middle, or high), birthweight status (<2500, 2500–3999, or ≥4000 g). ^c^ Multivariate model 2 was further adjusted for protein intake (% of energy, continuous), fat intake (% of energy, continuous), and dietary fiber intake (g/1000 kcal, continuous).

**Table 3:** Relationship of eating rate and degree of chewing with prevalence of being overweight among Japanese preschool children.

	Risk of Overweight ^a^						
	Overweight /Normal Weight (*n*)	Crude ^b^		Multivariate Model 1 ^c^		Multivariate Model 2 ^d^	
		OR	95% CI	OR	95% CI	OR	95% CI
Rate of eating							
Slow	117/1440	0.61	(0.48, 0.77)	0.55	(0.43, 0.71)	0.55	(0.43, 0.70)
Medium	198/1476	Reference		Reference		Reference	
Fast	149/433	2.57	(2.02, 3.26)	2.71	(2.11, 3.49)	2.71	(2.10, 3.48)
Effect per change in category		2.06	(1.80, 2.37)	2.22	(1.92, 2.57)	2.23	(1.93, 2.58)
*p* for trend		<0.001		<0.001		<0.001	
Degree of chewing							
Not well	168/657	2.12	(1.72, 2.62)	2.19	(1.75, 2.73)	2.18	(1.74, 2.73)
Medium	266/2208	Reference		Reference		Reference	
Well	30/484	0.52	(0.35, 0.76)	0.53	(0.36, 0.79)	0.53	(0.36, 0.79)
Effect per change in category		0.48	(0.41, 0.57)	0.48	(0.40, 0.57)	0.48	(0.40, 0.57)
*p* for trend		<0.001		<0.001		<0.001	

^a^ Overweight was defined according to the International Obesity Task Force cut-offs that are based on BMI [27]. ^b^ Values are odds ratio and 95% CIs in parentheses for being overweight against being normal weight. ^c^ Multivariate model 1 was adjusted for sex (boy or girl), age (months, continuous), residential block (Hokkaido and Tohoku; Kanto; Hokuriku and Tokai; Kinki; Chugoku and Shikoku; or Kyushu and Okinawa), paternal educational attainment (high school, junior college/vocational technical school, or university), maternal educational attainment (high school, junior college/vocational technical school, or university), paternal weight status (underweight, normal, or overweight), maternal weight status (underweight, normal, or overweight), number of siblings (0, 1, or ≥2), physical activity (low, middle, or high), birthweight status (<2500, 2500–3999, or ≥4000 g). ^d^ Multivariate model 2 was further adjusted for protein intake (% of energy, continuous), fat intake (% of energy, continuous), and dietary fiber intake (g/1000 kcal, continuous).

**Table 4:** Relationship of eating rate and degree of chewing with prevalence of thinness among Japanese preschool children.

	Risk of Thinness ^a^						
	Thinness/Normal Weight (*n*)	Crude ^b^		Multivariate Model 1 ^c^		Multivariate Model 2 ^d^	
		OR	95% CI	OR	95% CI	OR	95% CI
Rate of eating							
Slow	362/1440	1.59	(1.33, 1.90)	1.65	(1.37, 1.98)	1.63	(1.36, 1.98)
Medium	234/1476	Reference		Reference		Reference	
Fast	42/433	0.61	(0.43, 0.86)	0.58	(0.41, 0.83)	0.59	(0.41, 0.83)
Effect per change in category		0.63	(0.55, 0.72)	0.60	(0.52, 0.69)	0.60	(0.52, 0.69)
*p* for trend		<0.001		<0.001		<0.001	
Degree of chewing							
Not well	129/657	1.08	(0.87, 1.35)	1.05	(0.84, 1.31)	1.05	(0.84, 1.31)
Medium	400/2208	Reference		Reference		Reference	
Well	109/484	1.24	(0.98, 1.57)	1.26	(0.99, 1.60)	1.27	(0.99, 1.61)
Effect per change in category		1.06	(0.92, 1.23)	1.09	(0.94, 1.26)	1.09	(0.94, 1.26)
*p* for trend		0.42		0.26		0.26	

^a^ Thinness was defined according to the International Obesity Task Force cut-offs that are based on BMI [27]. ^b^ Values are odds ratio and 95% CIs in parentheses for being thin against being normal weight. ^c^ Multivariate model 1 was adjusted for sex (boy or girl), age (months, continuous), residential block (Hokkaido and Tohoku; Kanto; Hokuriku and Tokai; Kinki; Chugoku and Shikoku; or Kyushu and Okinawa), paternal educational attainment (high school, junior college/vocational technical school, or university), maternal educational attainment (high school, junior college/vocational technical school, or university), paternal weight status (underweight, normal, or overweight), maternal weight status (underweight, normal, or overweight), number of siblings (0, 1 or ≥2), physical activity (low, middle, or high), birthweight status (<2500, 2500–3999, or ≥4000 g). ^d^ Multivariate model 2 was further adjusted for protein intake (% of energy, continuous), fat intake (% of energy, continuous), and dietary fiber intake (g/1000 kcal, continuous).

## References

[1] World Health Organization (2016). Report of the Commission on Ending Childhood Obesity.

[2] Dietz W.H. (1994). Critical periods in childhood for the development of obesity. Am. J. Clin. Nutr..

[3] Lawlor D.A., Chaturvedi N. (2006). Treatment and prevention of obesity--are there critical periods for intervention?. Int. J. Epidemiol..

[4] Reilly J.J., Kelly J. (2011). Long-term impact of overweight and obesity in childhood and adolescence on morbidity and premature mortality in adulthood: Systematic review. Int. J. Obes..

[5] Umer A., Kelley G.A., Cottrell L.E., Giacobbi P. Jr., Innes K.E., Lilly C.L. (2017). Childhood obesity and adult cardiovascular disease risk factors: A systematic review with meta-analysis. BMC Public Health..

[6] Mikkilä V., Räsänen L., Raitakari O.T., Pietinen P., Viikari J. (2005). Consistent dietary patterns identified from childhood to adulthood: The cardiovascular risk in Young Finns Study. Br. J. Nutr..

[7] Nishtar S., Gluckman P., Armstrong T. (2016). Ending childhood obesity: A time for action. Lancet.

[8] Butland B., Jebb S., Kopelman P., McPherson K., Thomas S., Mardell J., Parry V. (2007). Tackling Obesities: Future Choices—Project Report. 2nd Edition. London: The Stationery Office. https://www.gov.uk/government/uploads/system/uploads/attachment_data/file/287937/07-1184x-tackling-obesities-future-choices-report.pdf.

[9] Ohkuma T., Hirakawa Y., Nakamura U., Kiyohara Y., Kitazono T., Ninomiya T. (2015). Association between eating rate and obesity: A systematic review and meta-analysis. Int. J. Obes..

[10] Lewellyn C.H., van Jaarsveld C.H., Boniface D., Carnell S., Wardle J. (2008). Eating rate is a heritable phenotype related to weight in children. Am. J. Clin. Nutr..

[11] Sun Y., Sekine M., Kagamimori S. (2009). Lifestyle and overweight among Japanese adolescents: The Toyama Birth Cohort Study. J. Epidemiol..

[12] Murakami K., Miyake Y., Sasaki S., Tanaka K., Arakawa M. (2012). Self-reported rate of eating and risk of overweight in Japanese children: Ryukyus Child Health Study. J. Nutr. Sci. Vitaminol. (Tokyo).

[13] Lin M., Pan L., Tang L., Jiang J., Wang Y., Jin R. (2014). Association of eating speed and energy intake of main meals with overweight in Chinese pre-school children. Public Health Nutr..

[14] Okubo H., Miyake Y., Sasaki S., Tanaka K., Hirota Y. (2017). Rate of eating in early life is positively associated with current and later body mass index among young Japanese children: The Osaka Maternal and Child Health Study. Nutr. Res..

[15] Fogel A., Goh A.T., Fries L.R., Sadananthan S.A., Velan S.S., Michael N., Tint M.T., Fortier M.V., Chan M.J., Toh J.Y. (2017). Faster eating rates are associated with higher energy intakes during an ad libitum meal, higher BMI and greater adiposity among 4·5-year-old children: Results from the Growing Up in Singapore Towards Healthy Outcomes (GUSTO) cohort. Br. J. Nutr..

[16] Andrade A.M., Greene G.W., Melanson K.J. (2008). Eating slowly led to decreases in energy intake within meals in healthy women. J. Am. Diet. Assoc..

[17] Zhu Y., Hollis J.H. (2014). Increasing the number of chews before swallowing reduces meal size in normal-weight, overweight, and obese adults. J. Acad. Nutr. Diet..

[18] Cassady B.A., Hollis J.H., Fulford A.D., Considine R.V., Mattes R.D. (2009). Mastication of almonds: Effects of lipid bioaccessibility, appetite, and hormone response. Am. J. Clin. Nutr..

[19] Fukuda H., Saito T., Mizuta M., Ishimatsu T., Nishikado S., Takagi H., Konomi Y. (2013). Chewing number is related to incremental increases in body weight from 20 years of age in Japanese middle-aged adults. Gerodontology.

[20] Ekuni D., Furuta M., Takeuchi N., Tomofuji T., Morita M. (2012). Self-reports of eating quickly are related to a decreased number of chews until first swallow, total number of chews, and total duration of chewing in young people. Arch. Oral. Biol..

[21] Zhu Y., Hollis J.H. (2015). Relationship between chewing behavior and body weight status in fully dentate healthy adults. Int. J. Food. Sci. Nutr..

[22] Ochiai H., Shirasawa T., Nanri H., Nishimura R., Nomoto S., Hoshino H., Kokaze A. (2012). Lifestyle factors associated with underweight among Japanese adolescents: A cross-sectional study. Arch. Public Health.

[23] Inokuchi M., Matsuo N., Takayama J.I., Hasegawa T. (2014). Trends in thin body stature among Japanese male adolescents, 2003-2012. Ann. Hum. Biol..

[24] Inokuchi M., Matsuo N., Takayama J.I., Hasegawa T. (2015). Trends in thin body stature among Japanese female adolescents, 2003-2012. Ann. Hum. Biol..

[25] Saido M., Asakura K., Masayasu S., Sasaki S. (2016). Relationship between Dietary Sugar Intake and Dental Caries among Japanese Preschool Children with Relatively Low Sugar Intake (Japan Nursery School SHOKUIKU Study): A Nationwide Cross-Sectional Study. Mater. Child Health J..

[26] Asakura K., Masayasu S., Sasaki S. (2017). Dietary intake, physical activity, and time management are associated with constipation in preschool children in Japan. Asia Pac. J. Clin. Nutr..

[27] Cole T.J., Lobstein T. (2012). Extended international (IOTF) body mass index cut-offs for thinness, overweight and obesity. Pediatr. Obes..

[28] World Health Organization (2000). Obesity: Preventing and Managing the Global Epidemic.

[29] Asakura K., Haga M., Sasaki S. (2015). Relative validity and reproducibility of a brief-type self-administered diet history questionnaire for Japanese children aged 3-6 years: Application of a questionnaire established for adults in preschool children. J. Epidemiol..

[30] Sasaki S., Yanagibori R., Amano K. (1998). Self-administered diet history questionnaire developed for health education: A relative validation of the test-version by comparison with 3-day diet record in women. J. Epidemiol..

[31] Sasaki S., Yanagibori R., Amano K. (1998). Validity of a self-administered diet history questionnaire for assessment of sodium and potassium: Comparison with single 24-h urinary excretion. Jpn. Circ. J..

[32] Kobayashi S., Murakami K., Sasaki S., Okubo H., Hirota N., Notsu A., Fukui M., Date C. (2011). Comparison of relative validity of food group intakes estimated by comprehensive and brief-type self-administered diet history questionnaires against 16 d dietary records in Japanese adults. Public Health Nutr..

[33] Kobayashi S., Honda S., Murakami K., Sasaki S., Okubo H., Hirota N., Notsu A., Fukui M., Date C. (2012). Both comprehensive and brief self-administered diet history questionnaires satisfactorily rank nutrient intakes in Japanese adults. J. Epidemiol..

[34] Science and Technology Agency (2010). Standard Tables of Food Composition in Japan 2010.

[35] Robinson E., Almiron-Roig E., Rutters F., de Graaf C., Forde C.G., Tudur S.C., Nolan S.J., Jebb S.A. (2014). A systematic review and meta-analysis examining the effect of eating rate on energy intake and hunger. Am. J. Clin. Nutr..

[36] Sasaki S., Katagiri A., Tsuji T., Shimoda T., Amano K. (2003). Self-reported rate of eating correlates with body mass index in 18-y-old Japanese women. Int. J. Obes. Relat. Metab. Disord..

[37] Kokkinos A., le Roux C.W., Alexiadou K., Tentolouris N., Vincent R.P., Kyriaki D., Perrea D., Ghatei M.A., Bloom S.R., Katsilambros N. (2010). Eating slowly increases the postprandial response of the anorexigenic gut hormones, peptide YY and glucagon-like peptide-1. J. Clin. Endocrinol. Metab..

[38] Birch L.L., Davison K.K. (2001). Family environmental factors influencing the developing behavioral controls of food intake and childhood overweight. Pediatr. Clin. N. Am..

[39] Gerace T.A., George V.A. (1996). Predictors of weight increases over 7 years in fire fighters and paramedics. Prev. Med..

[40] Ochiai H., Shirasawa T., Ohtsu T., Nishimura R., Morimoto A., Hoshino H., Tajima N., Kokaze A. (2014). The impact of eating quickly on anthropometric variables among schoolgirls: A prospective cohort study in Japan. Eur. J. Public Health.

[41] Ford A.L., Bergh C., Södersten P., Sabin M.A., Hollinghurst S., Hunt L.P., Shield J.P. (2009). Treatment of childhood obesity by retraining eating behaviour: Randomised controlled trial. BMJ.

[42] McGee T.L., Grima M.T., Hewson I.D., Jones K.M., Duke E.B., Dixon J.B. (2012). First Australian experiences with an oral volume restriction device to change eating behaviors and assist with weight loss. Obesity.

[43] Freedman D.S., Sherry B. (2009). The validity of BMI as an indicator of body fatness and risk among children. Pediatrics.

[44] Goodman E., Hinden B.R., Khandelwal S. (2000). Accuracy of teen and parental reports of obesity and body mass index. Pediatrics.

